# Evaluation of a novel method to assess corticosteroid responsiveness in chronic obstructive pulmonary disease

**DOI:** 10.4103/1817-1737.69114

**Published:** 2010

**Authors:** Fahad A Al-Ghimlas, Andrew McIvor

**Affiliations:** *Division of Respiratory Medicine, Department of Medicine, Amiri Hospital, Safat, Kuwait*; 1 *Department of Medicine, St. Joseph’s Healthcare Hamilton, Firestone Institute for Respiratory Health, Hamilton, Canada*

**Keywords:** COPD, corticosteroids, home spirometry, responsiveness, variability

## Abstract

**BACKGROUND:**

Some patients with chronic obstructive pulmonary disease (COPD) may benefit from oral steroid therapy. These steroid-responsive patients are diagnosed based on laboratory spirometry. We hypothesize that daily, home-based spirometry is a better tool.

**METHODS:**

Thirty patients with COPD underwent a single-blinded study, with a crossover design. They received 2 weeks of placebo followed by 2 weeks of prednisone therapy (40 mg/day). Laboratory spirometry was done at the beginning and end of the study and daily home-based spirometry was done twice a day.

**RESULTS:**

Analysis of variance model was used. The variability of the median day-to-day forced expiratory volume in 1 s (FEV_1_) was 72.5 mL (25th percentile of 40 mL and 75th percentile of 130 mL). The daily FEV_1_ variation was 70 mL (25th percentile of 50 mL and 75th percentile of 100 mL). The overall laboratory FEV_1_ variability was larger after the steroid course (*P* < 0.001), but not clinically significant. The variability was not significant postplacebo treatment compared with the baseline values. For home-based spirometry, steroid treatment was not significantly different. The majority (97%) completed more than 80% of the measurements. Ninety percent of the performed tests were considered acceptable. Only 53% of the tests were considered accurate. Overall both laboratory and home-based measurements did not show significant association between airway responsiveness and dyspnea or exercise capacity.

**CONCLUSION:**

Twice-daily home measurements of FEV_1_ might be better than the conventional approach to identify steroid responsive COPD patients. However, this finding was only statistically but not clinically significant. Therefore, we would not recommend this approach to identify COPD patients with steroid responsiveness.

Chronic obstructive pulmonary disease (COPD) based on the definition by the American Thoracic Society (ATS)[1] is a preventable and treatable disease state characterized by airflow limitation that is not fully reversible. At present, the guidelines for the treatment of COPD recommend detecting individuals who would benefit from steroids by conducting an oral steroid trial.[1] In their simplest form, physicians have been advised to administer an oral steroid trial daily for 2 weeks comparing laboratory-based measurements of breathing (spirometry) that are done in the beginning and the end of the trial, to determine whether there has been a significant response. This is defined in terms of change in the forced expiratory volume in 1 s (FEV_1_). A steroid responsiveness is characterized by an increase in the FEV_1_ by a certain level. This positive response suggests continuing the use of steroids, in the form of inhaled corticosteroids (ICS) or less likely oral steroids.

Recent research has cast doubt on the strategy of using an oral steroid trial to guide the management of individual patients with COPD. There have been more than 30 trials examining the effect of oral steroids in COPD. Methodologic concerns were noted in some of these trials. For example, some did not include a control group,[2–4] and others did not define the inclusion criteria adequately,[3,5] with the risk that patients with asthma may have been included. Later, better-designed trials showed a significant treatment effect with steroids. This effect was usually due to a marked improvement, for example, more than 50% increase in the FEV_1_, in a small group of patients, rather than a moderate improvement in the group overall.[6–10] In these trials with positive results, investigators have been unable to identify patient characteristics that would predict steroid responsiveness reliably.

In a meta-analysis by Calahan and colleagues, 10 studies of oral steroids in stable COPD were examined.[11] There was evidence that oral steroids reduced spirometric improvement in the FEV_1_ in approximately 10% of patients with stable COPD. Although such a finding might encourage physicians to look for steroid responsiveness in their patients with COPD, the results of the meta-analysis also suggest that the findings should be extrapolated to individual patients cautiously. There was a marked variability among the trials in the degree of steroid-responsiveness. To some degree, this may be accounted for by the differences in the patients recruited, disease definition, steroid dosing, and the study methodology.

Some small trials suggest that a few patients with stable COPD experience an unusually large FEV_1_ improvement following a 1- or 2-week course of systemic corticosteroids. Reference is sometimes made to COPD patients as being either “steroid-responsive” or “steroid-nonresponsive,” and the existence of these subtypes is implied in published guidelines for outpatient treatment of COPD. However, the ISOLDE study[12] failed to show a positive predictive value for the steroid trial in a large group of patients subsequently randomized to inhaled fluticasone in high dosage or inhaled placebo. Therefore, the use of steroids in COPD might be controversial.

Random fluctuations in breathing measurements could be mistaken for a positive response to therapy. Evidence of previous research indicates that steroid overuse may result from such random fluctuation being overinterpreted by physicians. The accuracy of the steroid trial might be improved by the use of repeated measurements throughout the period of steroid administration. In the past, it has been impractical and expensive to make twice or even daily measurement of lung function in the pulmonary function testing (PFT) laboratories. However, new handheld portable electronic devices can allow patients to perform this test themselves, at home, as frequently as needed to gauge responsiveness accurately.

The Canadian guidelines for the assessment and management of COPD[13] recommend assessing each patient’s response to steroids if airway obstruction and symptoms persist despite smoking cessation and optimum bronchodilator (BD) therapy. The steroid trial must be undertaken only at a time of clinical stability. During a maximum BD therapy, the patient will be given prednisone with a dose of 0.5 mg/kg for 2 or 3 weeks, after which PFT will be repeated. An improvement in the FEV_1_ of at least 20% and 200 mL will be regarded as steroid responsiveness. The ATS recommends in COPD patients with suboptimal control of symptoms that the physician should consider an oral steroid trial, for example, prednisone, up to 40 mg/day for 10–14 days.[1,14] If improvement occurs, the dosage should be lowered or alternate-day dose should be followed. If no improvement occurs, the steroid administration should be stopped. The positive steroid response is defined as an increase in the FEV_1_ ≥ 10% of the predicted value and/or >200 mL. The trial should be done in an exacerbation-free period, using a dose of 0.4-0.6 mg/kg for a duration of 2–4 weeks.

The present study is designed to test the usefulness of repeated home-based measurements to detect clinically important steroid responses among patients with stable COPD. The study also compares this method of treatment assessment with the current clinical method of measuring FEV_1_ before and after, pre and post, respectively, treatment. We hypothesize that twice-daily home measurements of FEV_1_ will be better than conventional clinic-based measurement of FEV_1_ to distinguish steroid responsive from steroid nonresponsive COPD patients.

## Methods

### Objectives

In this study, we are interested to determine the spirometric variability of patients with clinically stable COPD and to obtain the measurements using a hand-held electronic spirometer. We are also testing the hypothesis that stable COPD patients will be deemed steroid nonresponsive more often using daily FEV_1_ measurements than with laboratory’s pre- and post-BD FEV_1_ measurements alone. Assessing the association of variability with subjective and objective measurements of improvements is considered as well. In addition, we would like to explore the rate of adherence and technical inadequacy at home in using the home spirometer by the included patients.

### Study population

For this study, participants were drawn from outpatients attending the respiratory clinics at the Toronto Western Hospital (a division of the University Health Network, Ontario, Canada) between 2003 and 2004. Patients were included in the study if they had an established diagnosis of COPD according to the ATS criteria.[1] Each patient had to be an exsmoker for at least 1 year and with greater than 20 pack-year history of tobacco smoking. The participants also had to be clinically stable, which was defined as the absence of exacerbations within 3 months of enrolment. Finally, the participants were required to be symptomatic despite optimal BD therapy, to be included in the study.

Exclusion criteria included patients with major nonrespiratory diseases, such as congestive heart failure; chronic renal failure; diabetes mellitus or glucose intolerance; history of tuberculosis; use of steroid medication, oral or inhaled, 1 month prior to enrolment; or any known contraindication to the use of oral steroids. All the participants signed a written consent prior to their enrolment in the study.

After obtaining the research ethics board approval, the participants were identified and recruited for the study. The study protocol and rationale were explained to all of them, both verbally and in writing.

### The trial

The study has a single-blinded prospective placebo-control design of 30 stable COPD patients who received 2 weeks of placebo medication followed by 2 weeks of prednisone at a dose of 40 mg/day. To maintain blinding, the order of the placebo medication first and the oral steroid second was not outlined for the cardiopulmonary technologist performing the spirometry.

Spirometries pre- and post-BD (standard procedure) were performed in a laboratory setting before and after each treatment period. The participants were asked to abstain from inhaled BDs for 6 h and from sustained release theophylline for 48 h before the baseline spirometry. For spirometry repeated at the end of the placebo and prednisone treatment periods, only short-acting inhaled BDs were to be withheld before testing, and long-acting preparations were continued if they were part of the patients’ usual maintenance regimen. Post-BD testing was done 30 min after the administration of 4 puffs of inhaled salbutamol/ipratropium combination (Combivent, Boehringer Ingelheim Ltd, Canada) administered via adult aerochamber.

The participants were instructed by a research physician[Appendix A] about the use of a hand-held turbine-style electronic Diary Card^®^ (MicroMedical Diary Card, MicroMedical Ltd., Rochester, UK). This is a portable spirometer, which allows the patients to perform spirometry at home or anywhere outside the hospital. The device’s control unit has memory for several hundred spirometric recordings, including graphical storage of flow–volume curves (FVCs). The participants were instructed about spirometry for about 1 h, and they received written information on how to perform spirometry at home. After a learning period of 1 week, all the patients were able to produce technically acceptable flow–volume loops.

**Appendix A:** Instructions for the usage of Diary Card

1	Take a deep breath in.
2	Put the mouthpiece of the Diary Card into your mouth and make sure:
	You do not stick your tongue into the mouthpiece.
	Close your mouth tight so that air will not escape.
	Blow as hard as you can till you see 3 marks on the Diary Card.
3	Pull out the mouthpiece after the above procedure.
4	Read the instructions on the Diary Card and follow them.
5	Repeat the same procedure as above.
6	Blow 3 times at each occasion.
7	Repeat it 30 min after BD.
8	Repeat the same procedure in the evening twice (before and 30 min after the BD).

### Monitoring and follow-up

All the 30 patients were asked to use the Diary Cards 4 times a day at home during the 2 treatment periods of the trial. The expected number of spirometric measurements for a patient was 112, as 4 spirograms were done per day for a total duration of 28 days. The participants had to complete 4 follow-up visits. They were seen in the clinic at the beginning of week 1 (1^st^ visit for recruitment), beginning of week 2 (2^nd^ visit to start placebo), beginning of week 4 (3^rd^ visit to start prednisone), and at the end of the study after week 5.

### Outcome measures

In the 2^nd^ visit, baseline PFT took place in a hospital laboratory. The PFT included the measurements of height and weight, to calculate the body mass index. The participants were also instructed about using the Borg Scale[15] to report their symptom perception. In addition, assessment of the baseline dyspnea index (BDI)[16] and 6-min walk test (6MWT)[17] took place during the visit. Home-based spirometry was to be performed before (pre-BD) and 30 min after inhalation of BD (post-BD) every morning (AM) and evening (PM) using the Diary Card. The results reported are the highest FEV_1_ and forced vital capacity (FVC). To be considered technically acceptable, the test had to be performed correctly by inspection of the curve, and the chosen values should not exceed the next highest by more than 5%. Each participant used the same Diary Card for both periods of treatment. To obtain a true measure of the baseline pulmonary function, only the pre-BD home-based measurements of FEV_1_ during the placebo treatment period were used. The day-to-day variability was obtained by calculating the difference in home-based FEV_1_ (only AM measurements) compared with the first day value. The daily FEV_1_ variation was obtained by comparing the differences of FEV_1_ AM–PM measurements with the one presented on the first day of the placebo treatment. The participants were re-assessed to measure their transitional dyspnea index (TDI)[16] at the end of both placebo and steroid trials. The 6MWT was also measured at the end of each study trial. In the last visit, another hospital laboratory PFT was done. The participants’ reports of the Borg Scale were collected at the end of the study period.

### Adherence, acceptability, and adequacy

The subjects’ adherence with the use of the Diary Card was calculated using the formula:

$$\mathrm{Adherence}\;=\;\left(\mathrm{Number}\;\mathrm{of}\;\mathrm{Tests}\;\mathrm{Performed}\;\times \;100\right)/\mathrm{Total}\;\mathrm{Expected}\;\mathrm{Number}$$

The FEV_1_ readings obtained from the Dairy Card measurements were analyzed for quality control according to 2 criteria: the appearance of the graphic record of the FVC and the arithmetic reproducibility criteria. A spirogram was considered *acceptable* if it was free from artifacts (see Data handling section), and had a good start and a satisfactory exhalation. The percentage of *acceptable* spirometric recordings was calculated using the formula:

$$\mathrm{Percentage}\;\mathrm{of}\;\mathrm{acceptable}\;\mathrm{spirograms}\;=\;\left(\mathrm{Number}\;\mathrm{of}\;\mathrm{Acceptable}\;\mathrm{Spirograms}\;�\;100\right)/\mathrm{Total}\;\mathrm{Number}\;\mathrm{of}\;\mathrm{Performed}\;\mathrm{Spirograms}$$

A spirogram was considered *adequate* if it was considered technically *acceptable* and the chosen values did not exceed the next highest by more than 5%. The percentage of *adequate* spirometric recordings was calculated using the formula:

$$\mathrm{Percentage}\;\mathrm{of}\;\mathrm{adequate}\;\mathrm{spirograms}\;=\;\left(\mathrm{Number}\;\mathrm{of}\;\mathrm{Adequate}\;\mathrm{Spirograms}\;\times \;100\right)/\mathrm{Total}\;\mathrm{Number}\;\mathrm{of}\;\mathrm{Performed}\;\mathrm{Spirograms}$$

### Data handling

Patient-generated data (results of home spirometry) were analyzed for quality control before statistical analysis. Quality control of the Diary Card took place in the first 4 visits. All the flow–volume loops recorded were assessed. Using customary criteria, the loops were excluded if the graphic record suggested inadequate effort or improper technique.

### Sample size

We assumed a variance of 0.3, that is, a standard deviation (SD) of 0.55, based on the findings of several clinical trials.[4,6,7,18] Thirty participants were required to achieve an 80% power and a 20% difference in the FEV_1_ using this design. The latter percentage was considered the minimal clinically significant difference in the FEV_1_ for a responder to steroid treatment.[1] Only a change in the post-BD FEV_1_ was examined for this difference.

### Statistical techniques

The data were tested for normality and equal variance. For normally distributed data, paired t test was performed, reporting the mean and SD. For nonnormally distributed data, nonparametric Wilcoxon signed rank test was used, reporting the median and the 25^th^ and 75^th^ percentile points. Comparisons were made with a significance level of 0.05.

The analysis of variance was used to assess the reliability of the FEV_1_ measurements. General linear regression model was built to assess the association between the variability of FEV subjectively (using Borg Scale and dyspnea index measurements) and objectively (using 6MWT).

All data were analyzed with a database and statistical package (SigmaStat; Jandel Scientific, San Raphael, CA).

## Results

Thirty participants were recruited for the study, 14 males and 16 females. The mean age of the men and women was 70.0 and 68.3 years, respectively. The mean initial FEV_1_ in the male group was 1.15 L (37.7% of the predicted value), and it was 1.15 L (54.7% of the predicted value) in the female group.

**Figure 1:**
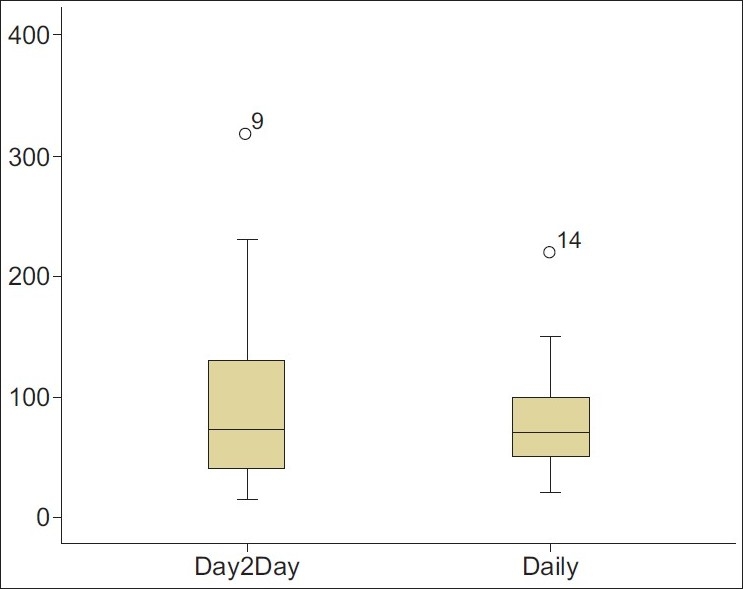
Box plot showing data distribution of both day-to-day (Day2Day) and daily variability of FEV1 during spirometric measurements

The variability of the median day-to-day FEV_1_ was 72.5 mL (3.0% of the predicted value). The 25^th^ and 75^th^ percentiles were 40 and 130 mL, respectively. The daily FEV_1_ variation was 70 mL (4.1% of the predicted value) with a 25th percentile of 50 mL and 75th percentile of 100 mL [Figure 1].

The overall variability in spirometric measurements was found to be larger than expected from random variability in the treatment, and therefore the treatments had different effects than would be expected by chance (*P* < 0.001). Furthermore, there was a statistically significant difference between the FEV_1_ values poststeroid treatment compared with both placebo treatment and baseline. A nonstatistically significant difference was found for the FEV_1_ values postplacebo treatment compared with the baseline values.

For the laboratory testing, FEV_1_ measurements poststeroid treatment were compared with those of postplacebo treatment when evaluating the efficacy of the oral steroid trial [Table 1]. There was a statistically significant difference for laboratory measurements after the 2 types of treatment.

**Table 1:** Results of the comparison of post-BD FEV_1_ laboratory measurements

Group	Median	25%	75%
Post-placebo	1.13	0.90	1.54
Post-steroid	1.21	0.99	1.76

The change that occurred with the treatment is greater than that would be expected by chance; there is a statistically significant difference (*P* < 0.001)

For home-based spirometry, steroid treatment was not significantly different [Table 2] for Diary Card measurements of FEV_1_. There was no significant difference between the responsive and nonresponsive groups.

**Table 2:** Results of the comparison of post-BD FEV_1_ Diary Card measurements

Group	Median	25%	75%
Post-placebo	1.08	0.79	1.30
Post-steroid	1.06	0.76	1.33

The change that occurred with the treatment is not great enough to exclude the possibility that it is due to chance (*P* = 0.86)

Both conventional and Diary Card measurements did not show significant association between airway responsiveness to steroids and symptoms of dyspnea assessed by Borg Scale, BDI, and dyspnea index measurements. The Borg Scale percentage of decrease in the mean of the baseline rating, after oral steroid or placebo trial, for responders was 22.51%, whereas it was 18.02% for nonresponders (*P* = 0.71). The BDI/TDI ratios for responders and nonresponders were 1.12 and 0.7, respectively (*P* = 0.09). However, 6MWT distance was associated significantly with airway responsiveness in participants using the Diary Card measurements with a mean percentage of increase in the walk distance, after oral steroid or placebo trial, of 13.3% among responders and 5.74% among nonresponders (*P* < 0.01) [Table 3].

**Table 3:** Comparison of responders and nonresponders, according to the Diary Card Measurements

Outcome	Mean % responders	Mean % Nonresponders	Mean % Nonresponders	*P* value
Borg scale	22,52	28.02	*t* test	0.71
BDI/TDI	1.12	0.7	Wilcoxon signed rank test	0.09
6MWT	13.3	5.74	*t* test	< 0.01

BDI = Baseline dyspnea index, TDI = Transitional dyspnea index, BDI/TDI = Ratio between BDI and TDI, 6MWT = 6-min walk test, *For normally distributed data, paired t test was performed. For nonnormally distributed data, nonparametric Wilcoxon signed rank test was used (see Statistical Techniques section)

The percentage of performed tests was calculated for each participant. The majority (29 participants, 97%) completed more than 80% of the measurements. Ninety percent of performed tests were considered acceptable. However, only 16 participants (53%) met the criteria for adequacy according to their test performance.

## Discussion

In this study, a new method of assessing the response in stable COPD patients to a short trial of oral steroid was assessed. Our hypothesis was that twice-daily home measurements of FEV_1_ would be better than conventional clinic-based measurements of FEV_1_ to distinguish steroid responsive COPD patients from steroid nonresponsive COPD patients. The design of the study and the criteria used to assess the responsiveness to steroids are according to the existing ATS guidelines at the time when the study began.

The inclusion criteria limited the possibility of confounders. All the patients had a smoking history of at least 20 pack-years, were exsmokers for at least 1 year, had a stable disease, and their FEV_1_ was less than 80% of the predicted value. Therefore, we specifically designed the study so no asthmatic patients would be included as possible. In addition, the inclusion criteria were chosen to eliminate potential biases related to smoking status and degree. By excluding recent exacerbations, we intended to prevent a potential bias as such patients would be more susceptible to respond to steroids than patients with a stable disease.

A major advantage of crossover studies is that they usually require smaller sample size than parallel group trials, as patients form their own controls. The reason for not randomizing the order of treatment was the uncertainty regarding the “wash-out” duration and the risk of steroids’ carried-over effect. A 2-week treatment period was used as it is felt that maximum improvement in airway obstruction, treated with steroids is achieved in approximately 8 days.[19]

It was known from other studies that the variability in the FEV_1_ is increased in patients with obstructive lung disease. Since this study is the first one to use repeated measurements of FEV_1_ in COPD patients, one of the objectives was to determine the spirometric variability in these participants while they have a stable disease. In our study participants, the day-to-day and daily variability of FEV_1_ was much lower than the values mentioned in other studies conducted on COPD patients.

We showed that laboratory measurements have a statistically significant response to steroids, whereas the daily home-based measurements did not. However, the size of improvement in the FEV_1_ represents a lower value than the clinically significant, since the difference found was less than 200 mL. This could be explained by the differences in the variability of FEV_1_ calculated using laboratory or home (Diary Card) measurements. To detect a smaller change in the FEV_1_ than planned, a bigger sample size would be needed given the assumed variance in the sample size formula. It is probable, however, that the real variance is smaller than the one chosen for the sample size. If the variance used for the sample size calculation was the same as the one found in our data, there would be no statistically significant difference. However, one of the objectives was to prove that FEV_1_ would be less variable using the Diary Card measurements. Higher variability of FEV_1_ measurements in the laboratory could appear to have a statistically significant difference after the steroid treatment. If the Diary Card measurements were more consistent, there would be less variation of FEV_1_, and so less frequent false-positive responses to steroids could be detected.

The hypothesis that twice-daily home measurements of FEV_1_ is better than the conventional clinic-based measurement of FEV_1_ to distinguish steroid responsive from steroid nonresponsive COPD patients was validated only from the statistical point of view (*P* < 0.005). The differences in the FEV_1_ were not clinically significant as the absolute difference was less than what was considered *a priori* a significant threshold for an acceptable steroid responsiveness, that is, 200 mL.

Eighty-three percent of the enrolled participants performed more than 91% of the required 112 home spirometry measurements. This means that they were adherent to the study intervention. Previous data[20] concluded that Diary Card machine could be used to monitor asthmatic patients. Ninety-two percent of our subjects had performed correct FEV_1_ home measurements according to the aspect of the graphic record criteria. This proves that the Diary Card machine could be used for monitoring PFTs for COPD patients as well. A much smaller number of corrected recordings according to both criteria (graphic and 5% value) could be explained by the absence of the technicians and their coaching ability during the home spirometry performance. With more training and may be frequent check-up of the quality of the recordings, the percentage of correct recordings could be improved. This might be achieved by increasing the frequency of clinic visits to once a week for each treatment period instead of only at the beginning and the end of treatment.

We recognize some limitations in our study. The study has a “fixed” crossover design and therefore was not randomized. This may limit the internal validity of the trial. However, we attempted to improve this limitation by blinding both participants and outcome assessors. Another limitation, also related to the study design, is the small sample size. In order to improve inferences and the external validity of the study to be more generalizable, a larger sample size might be required.

## Future Research

Many existing COPD guidelines[1,13] still recommend the use of a short course (2 weeks) of oral steroids to identify patients who might benefit from long-term treatment with inhaled or even oral steroids. On the other hand, the Global Initiative for Chronic Obstructive Lung Disease (GOLD) guidelines[21] recommend a trial of 6 weeks to 3 months with ICS, to identify COPD patients who may benefit from long-term inhaled steroid therapy. In addition, one of the GOLD new directions for future research is to develop and evaluate in clinical practice other measures than laboratory spirometry to assess and monitor COPD. Since 2 of the goals of effective COPD management are to relieve the symptoms and to improve exercise tolerance, the repeated measurements of FEV_1_ using Diary Card at home could be used in a future study. This could be done to assess the response to inhaled steroids.

Since the GOLD guidelines use 15% increase in the FEV_1_ above baseline as a criterion for steroid response instead of 20% used in our study, a bigger sample size would be needed for a future study. The results of our study allow calculating an estimate of the variance of the variability of single measurements on these participants. This estimate may be used to determine the required sample size in the subsequent study.

Our method to assess steroid responsiveness that may lead to a reduction in prescribing excessive steroids in COPD could have significant impact on provincial and national health care costs. This also needs to be explored in further research.

Finally, since inhaled steroids have fewer contraindications than oral steroids, the inclusion criteria for a following study should not be strict as the ones used in this study. This means that more eligible participants for the study could be allocated in a limited timeframe. In this situation, a crossover design with a randomization of 2 treatments (placebo and inhaled steroids) could be more feasible than in the present study.

## Conclusion

This is the first study demonstrating that twice-daily home measurements of FEV_1_ are better than the conventional clinic-based measurement of FEV_1_ to distinguish steroid responsive from steroid nonresponsive COPD patients. However, this finding was statistically but not clinically significant. Therefore, we would not recommend this approach to identify COPD patients with steroid responsiveness. Further research is needed in this area to explore further aspects of this interesting clinical problem.
